# Supplementary figures and images for: Dense GM-CSFRα-expressing immune infiltration is allied with longer survival of intrahepatic cholangiocarcinoma patients
Source: PeerJ. 2023 Mar 2;11:e14883. doi: 10.7717/peerj.14883 (PMC9985900; doi:10.7717/peerj.14883)

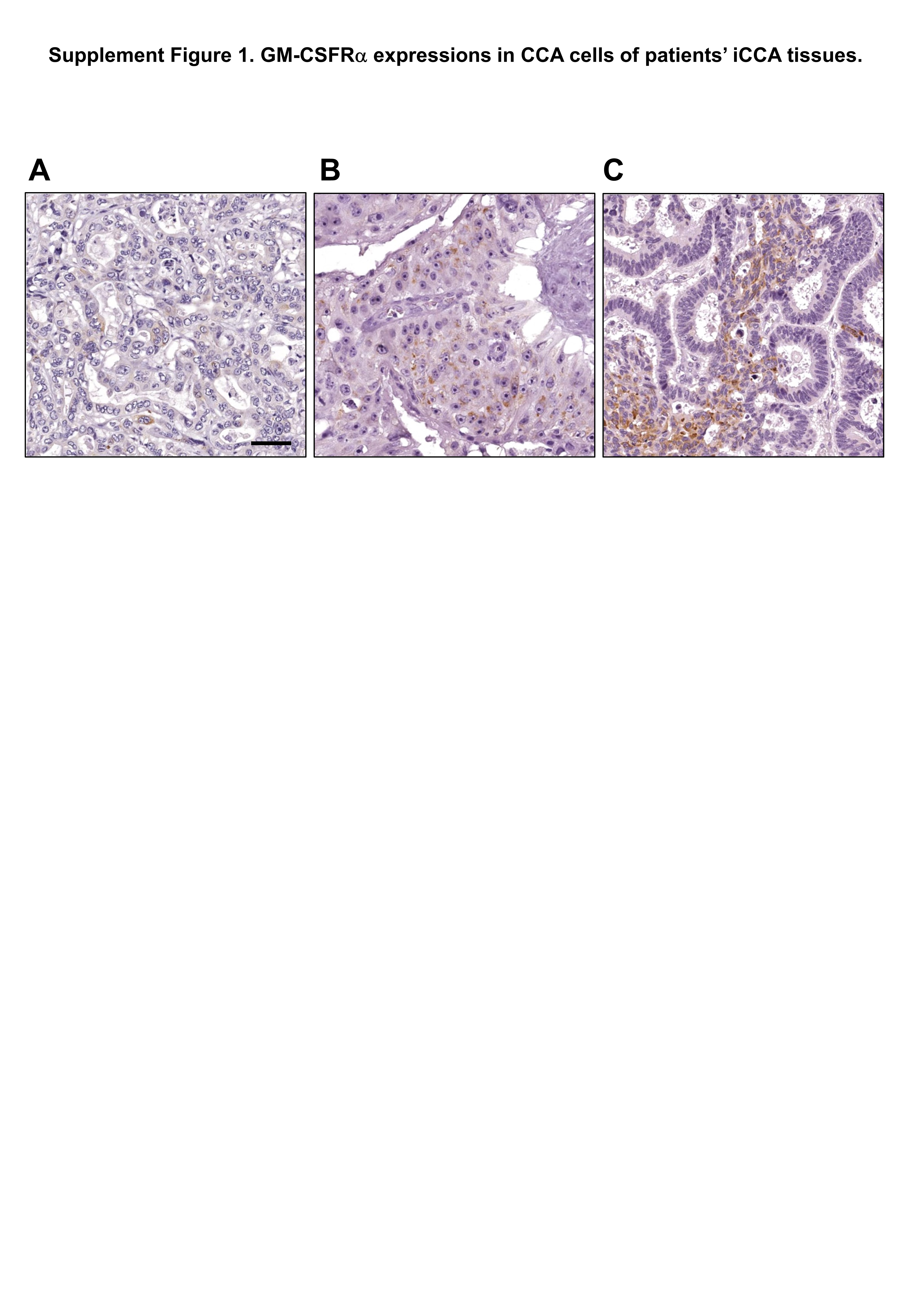

Supplement: Supplemental Information 1 — The immunohistochemistry of GM-CSFRα expression in CCA cells in non-papillary (A–B) and papillary subtypes (C). Bar = 50 µm. [file peerj-11-14883-s001.jpg]

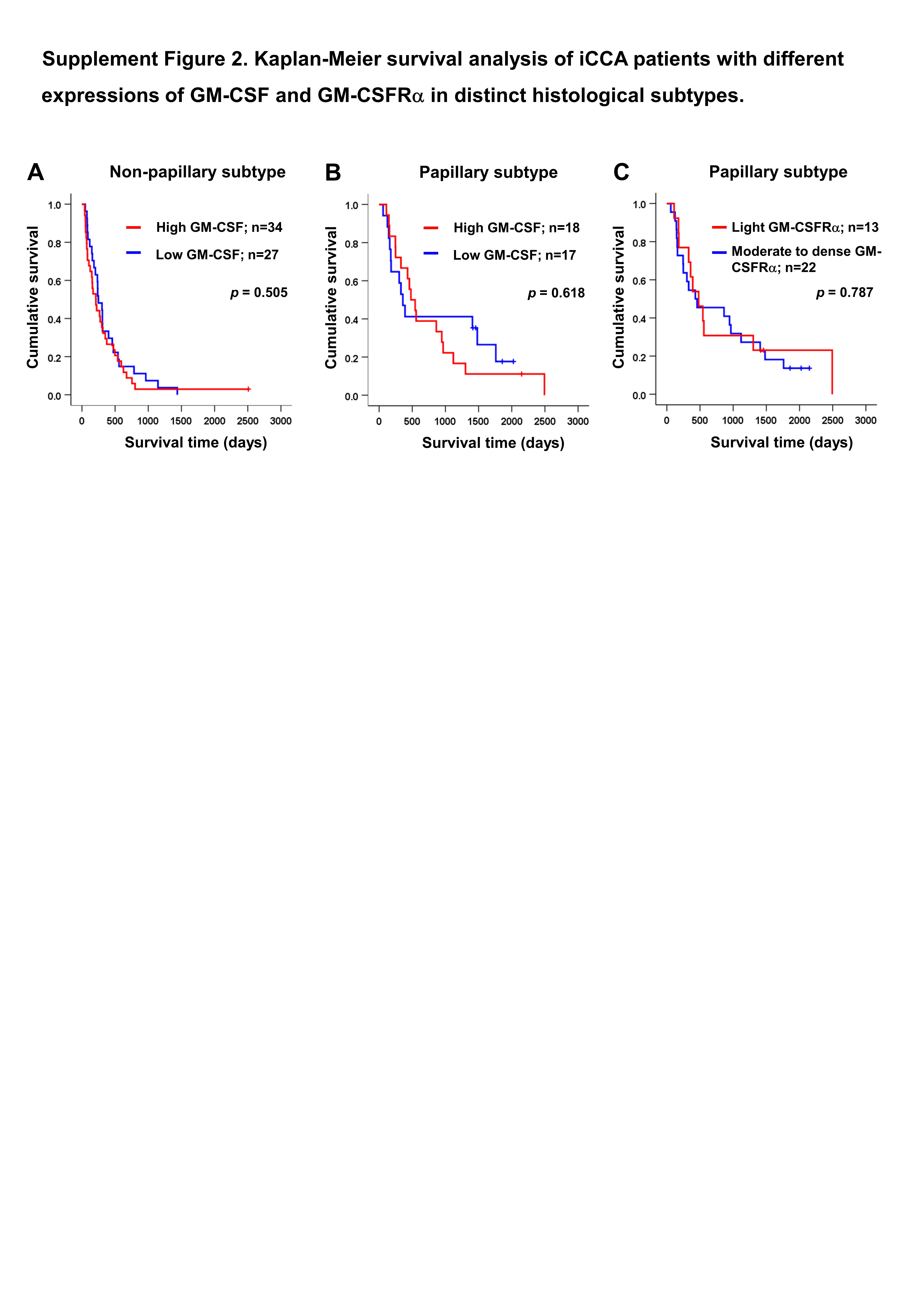

Supplement: Supplemental Information 2 — Kaplan–Meier survival analysis of different GM-CSF expressions in non-papillary (A) and papillary subtypes (B) of iCCA, and GM-CSFRα expressions in papillary subtypes (C). [file peerj-11-14883-s002.jpg]
