# Supplementary material for: Dense GM-CSFRα-expressing immune infiltration is allied with longer survival of intrahepatic cholangiocarcinoma patients
Source: PeerJ. 2023 Mar 2;11:e14883. doi: 10.7717/peerj.14883 (PMC9985900; doi:10.7717/peerj.14883)
Supplement: Supplemental Information 3 [file peerj-11-14883-s003.pdf]

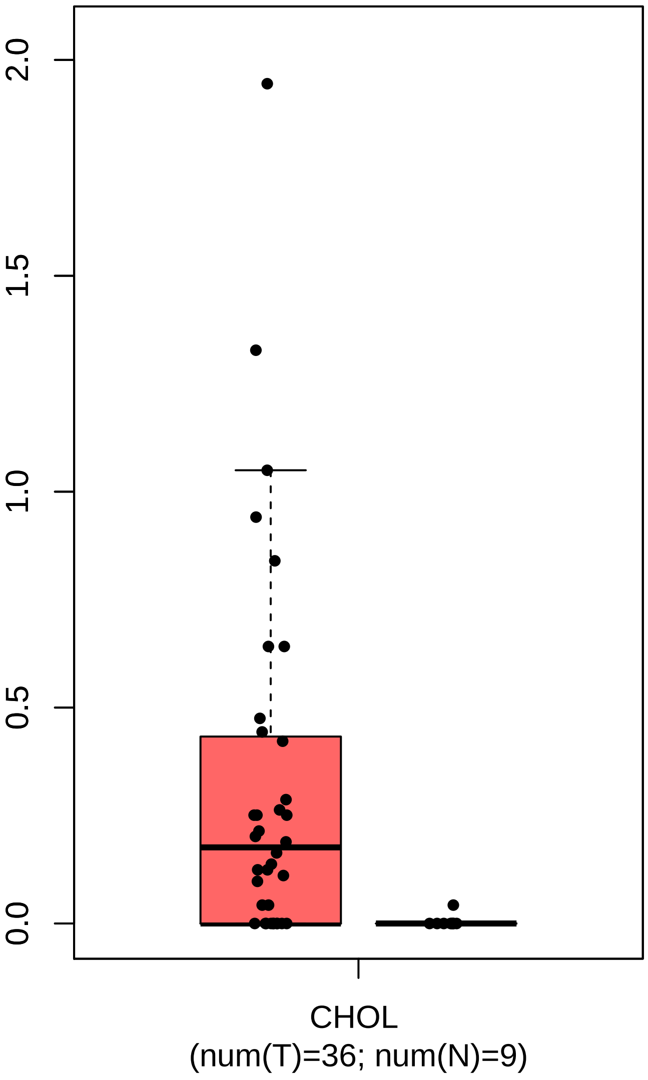

2.0
1.5
1.0
0.5
0.0
CHOL
(num(T)=36; num(N)=9)

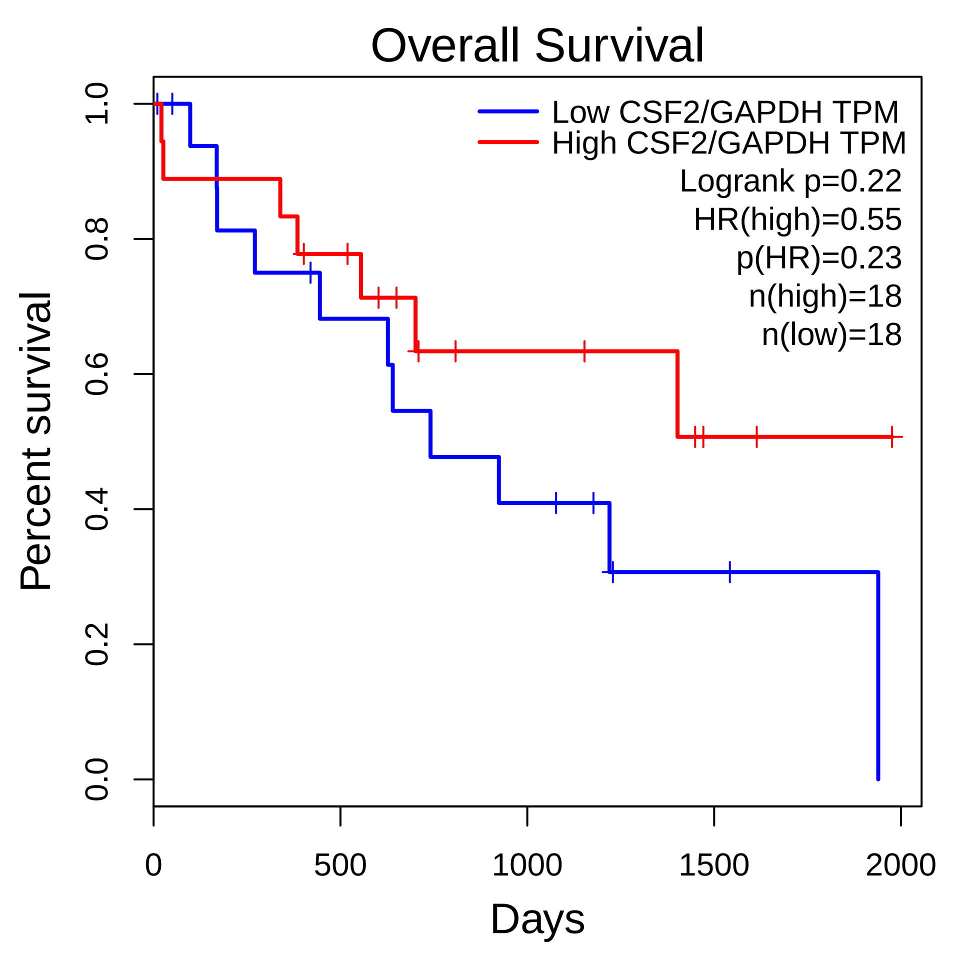
Overall Survival
Low CSF2/GAPDH TPM
High CSF2/GAPDH TPM
Logrank p=0.22
HR(high)=0.55
p(HR)=0.23
n(high)=18
n(low)=18
Percent survival
1.0
0.8
0.6
0.4
0.2
0.0
0
500
1000
1500
2000
Days

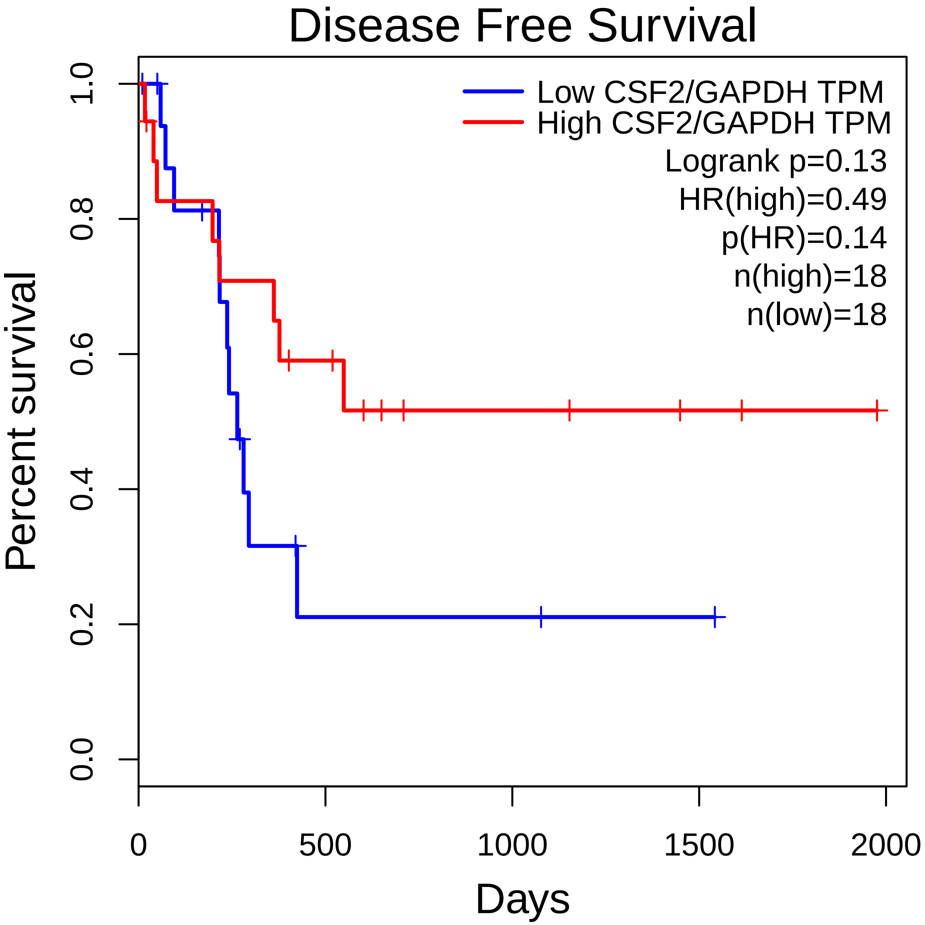

Disease Free Survival
Low CSF2/GAPDH TPM
High CSF2/GAPDH TPM
Logrank p=0.13
HR(high)=0.49
p(HR)=0.14
n(high)=18
n(low)=18
Percent survival
0.0
0.2
0.4
0.6
0.8
1.0
0
500
1000
1500
2000
Days

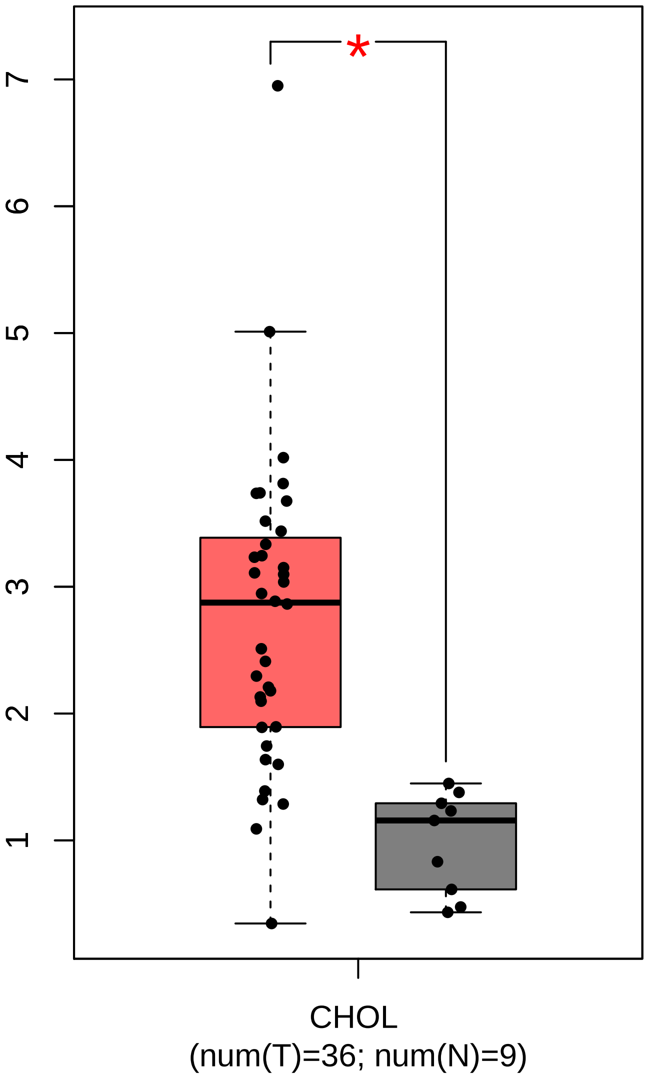

*
1
2
3
4
5
6
7
CHOL
(num(T)=36; num(N)=9)

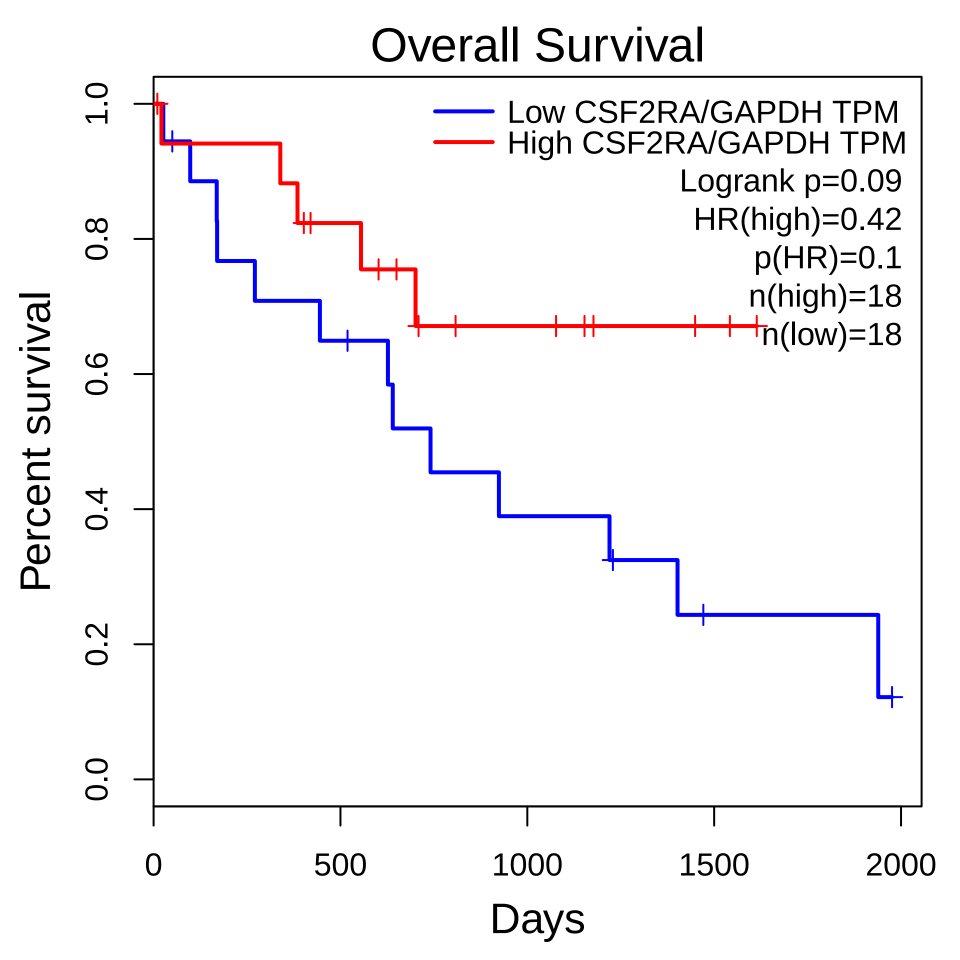

Overall Survival
Low CSF2RA/GAPDH TPM
High CSF2RA/GAPDH TPM
Logrank p=0.09
HR(high)=0.42
p(HR)=0.1
n(high)=18
n(low)=18
Percent survival
0.0
0.2
0.4
0.6
0.8
1.0
0
500
1000
1500
2000
Days

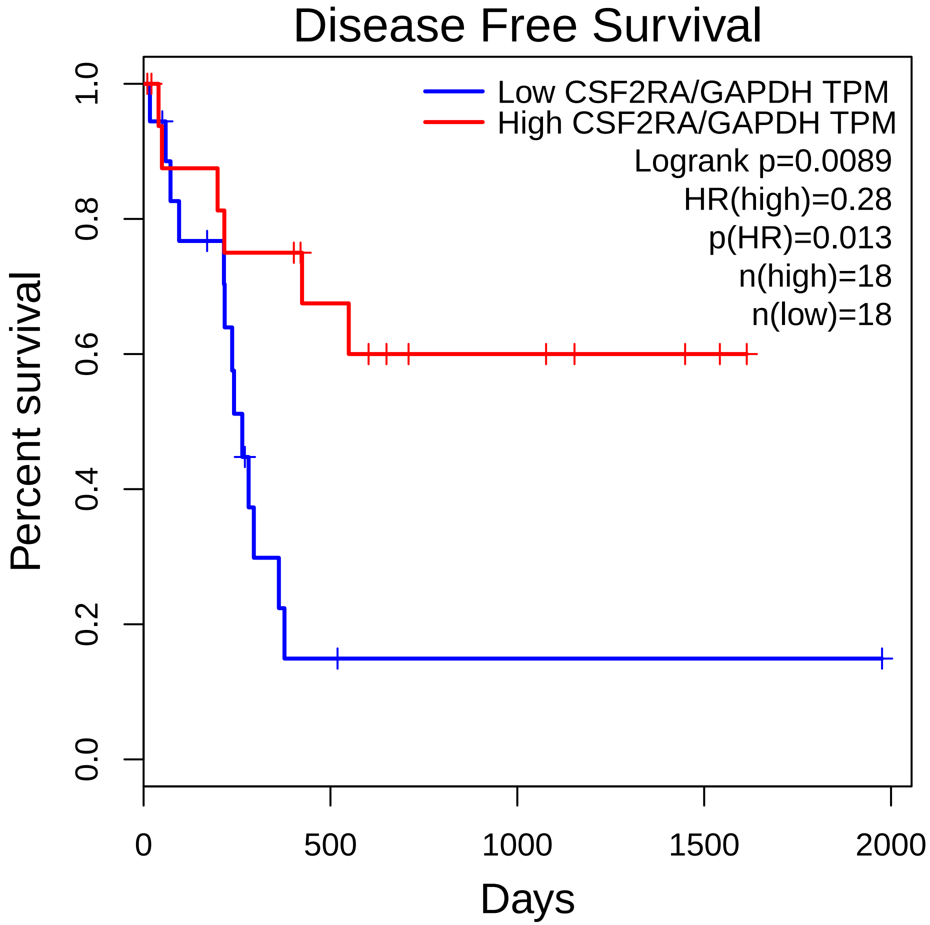
Disease Free Survival
Low CSF2RA/GAPDH TPM
High CSF2RA/GAPDH TPM
Logrank p=0.0089
HR(high)=0.28
p(HR)=0.013
n(high)=18
n(low)=18
Percent survival
0.0
0.2
0.4
0.6
0.8
1.0
0
500
1000
1500
2000
Days
