# Supplementary material for: Dense GM-CSFRα-expressing immune infiltration is allied with longer survival of intrahepatic cholangiocarcinoma patients
Source: PeerJ. 2023 Mar 2;11:e14883. doi: 10.7717/peerj.14883 (PMC9985900; doi:10.7717/peerj.14883)
Supplement: Supplemental Information 10 [file peerj-11-14883-s010.pdf]

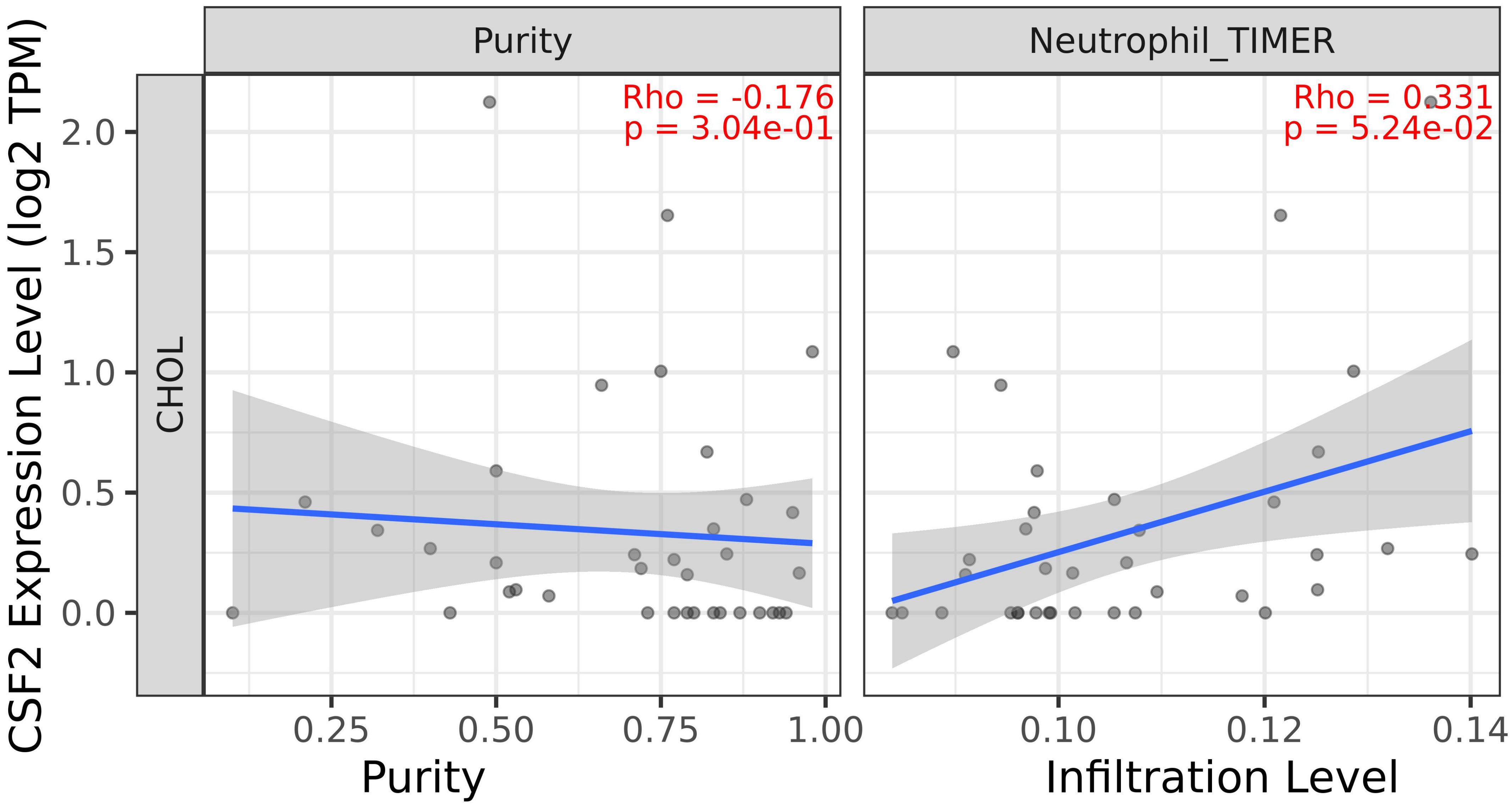

Purity
Neutrophil_TIMER
CHOL
CSF2 Expression Level (log2 TPM)
Rho = -0.176
p = 3.04e-01
Rho = 0.331
p = 5.24e-02
2.0
1.5
1.0
0.5
0.0
0.25
0.50
0.75
1.00
0.10
0.12
0.14
Purity
Infiltration Level

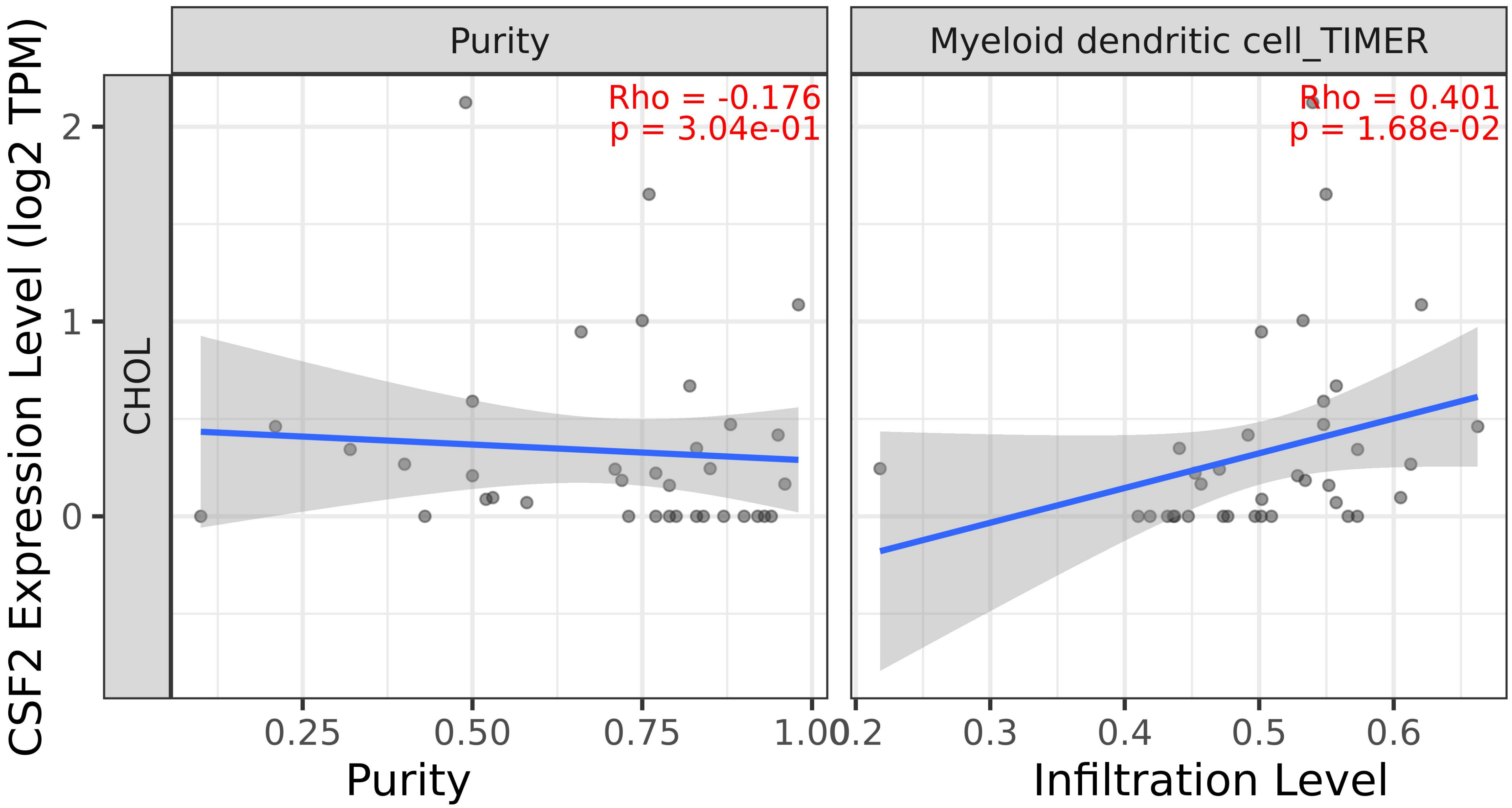

Purity
Myeloid dendritic cell_TIMER
Rho = -0.176
p = 3.04e-01
Rho = 0.401
p = 1.68e-02
CHOL
CSF2 Expression Level (log2 TPM)
2
1
0
0.25
0.50
0.75
1.00
0.2
0.3
0.4
0.5
0.6
Purity
Infiltration Level

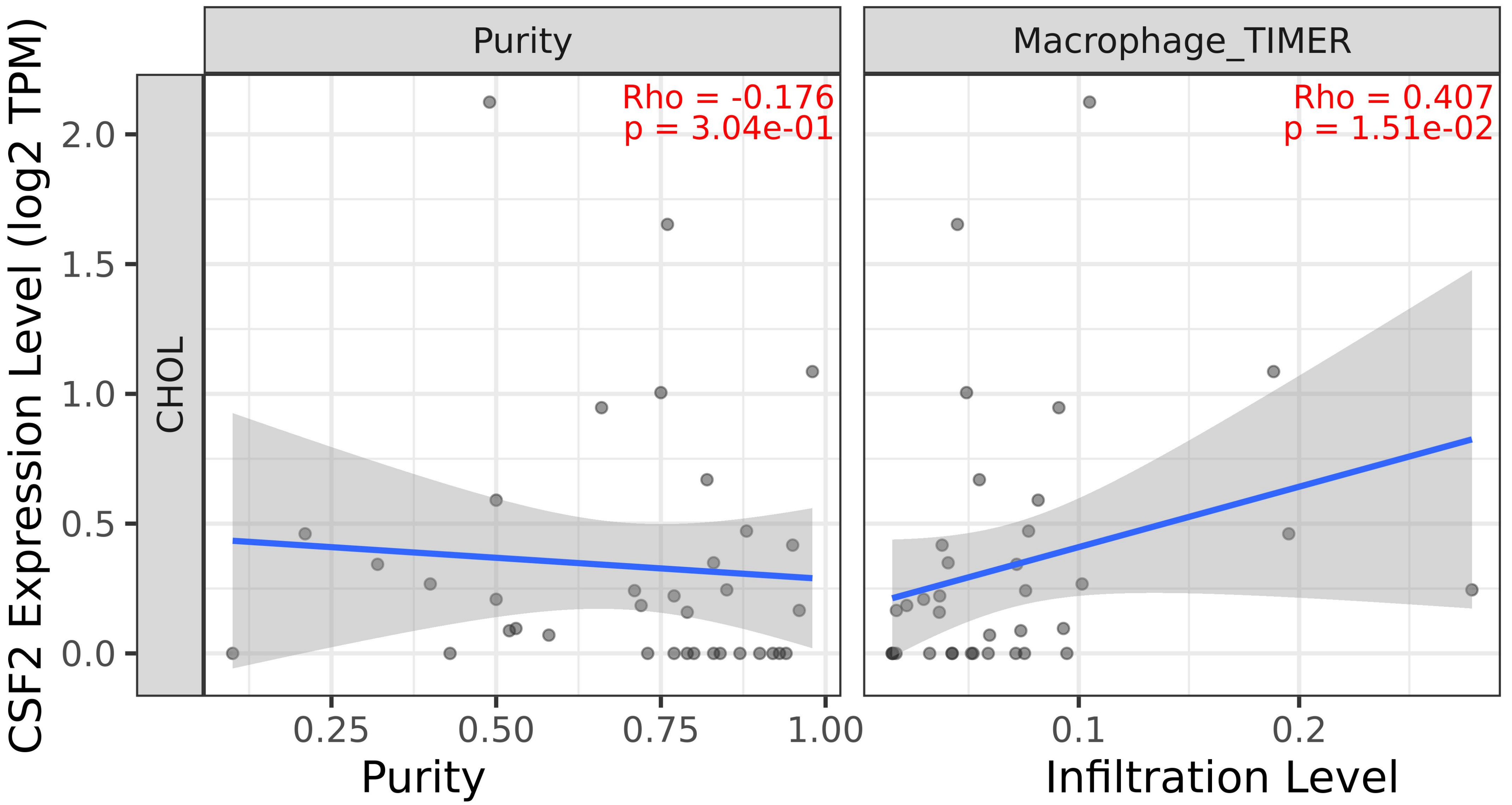

Purity
Macrophage_TIMER
CHOL
Rho = -0.176
p = 3.04e-01
Rho = 0.407
p = 1.51e-02
CSF2 Expression Level (log2 TPM)
2.0
1.5
1.0
0.5
0.0
0.25
0.50
0.75
1.00
0.1
0.2
Purity
Infiltration Level

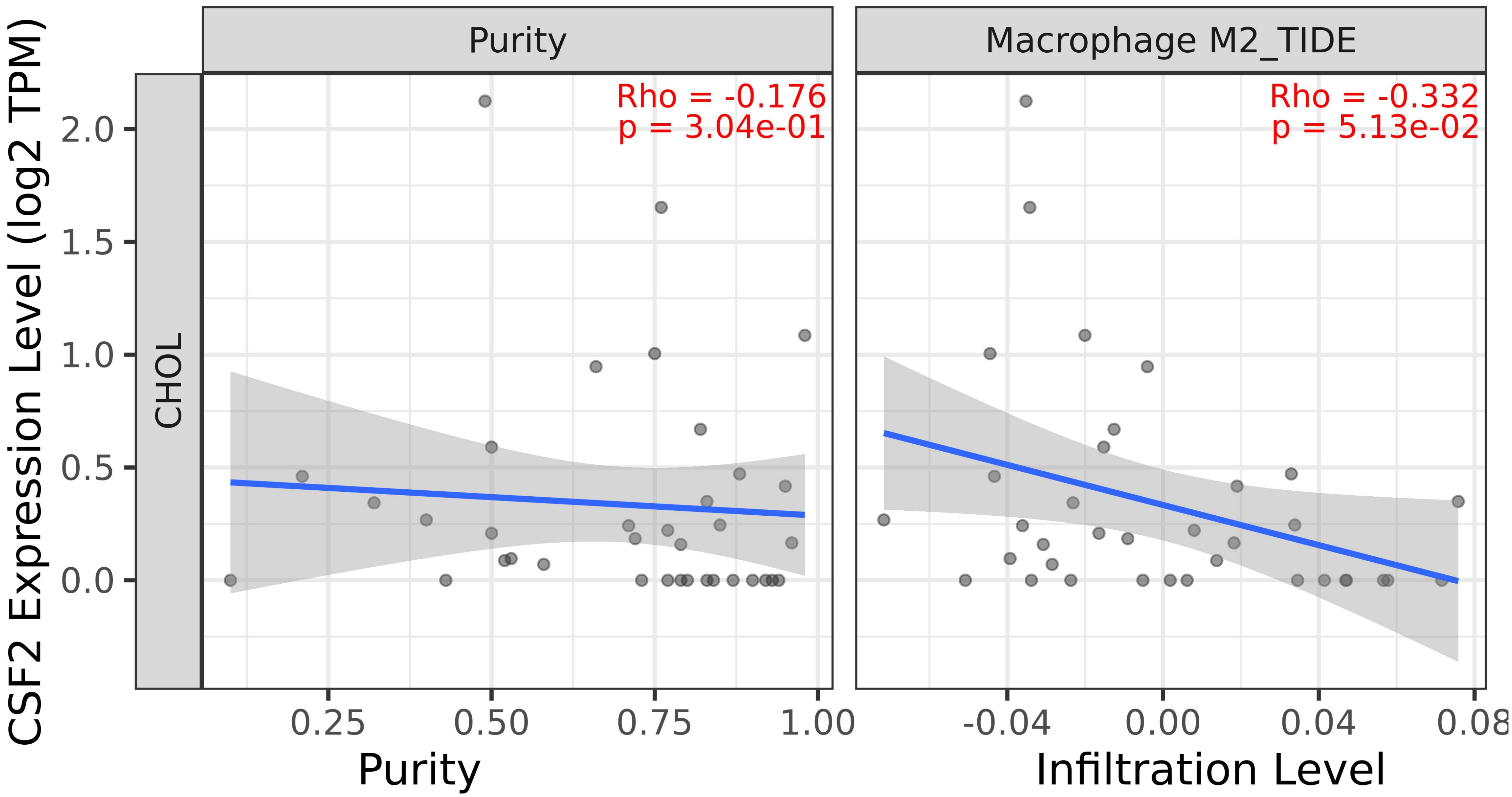

Purity
Macrophage M2_TIDE
CHOL
CSF2 Expression Level (log2 TPM)
Rho = -0.176
p = 3.04e-01
Rho = -0.332
p = 5.13e-02
2.0
1.5
1.0
0.5
0.0
0.25
0.50
0.75
1.00
-0.04
0.00
0.04
0.08
Purity
Infiltration Level

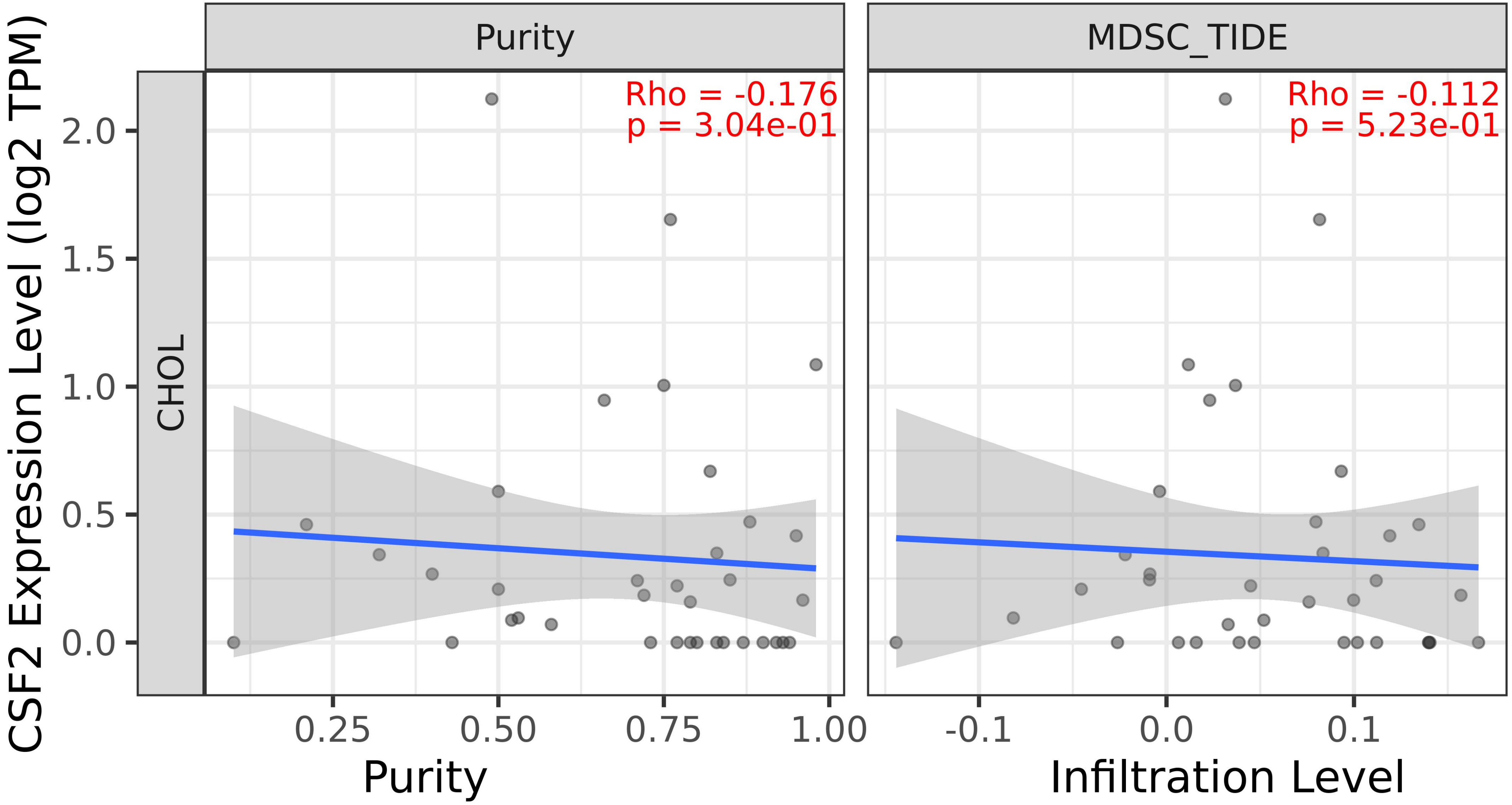
Purity
MDSC_TIDE
CHOL
Rho = -0.176
p = 3.04e-01
Rho = -0.112
p = 5.23e-01
CSF2 Expression Level (log2 TPM)
0.0
0.5
1.0
1.5
2.0
0.25
0.50
0.75
1.00
-0.1
0.0
0.1
Purity
Infiltration Level

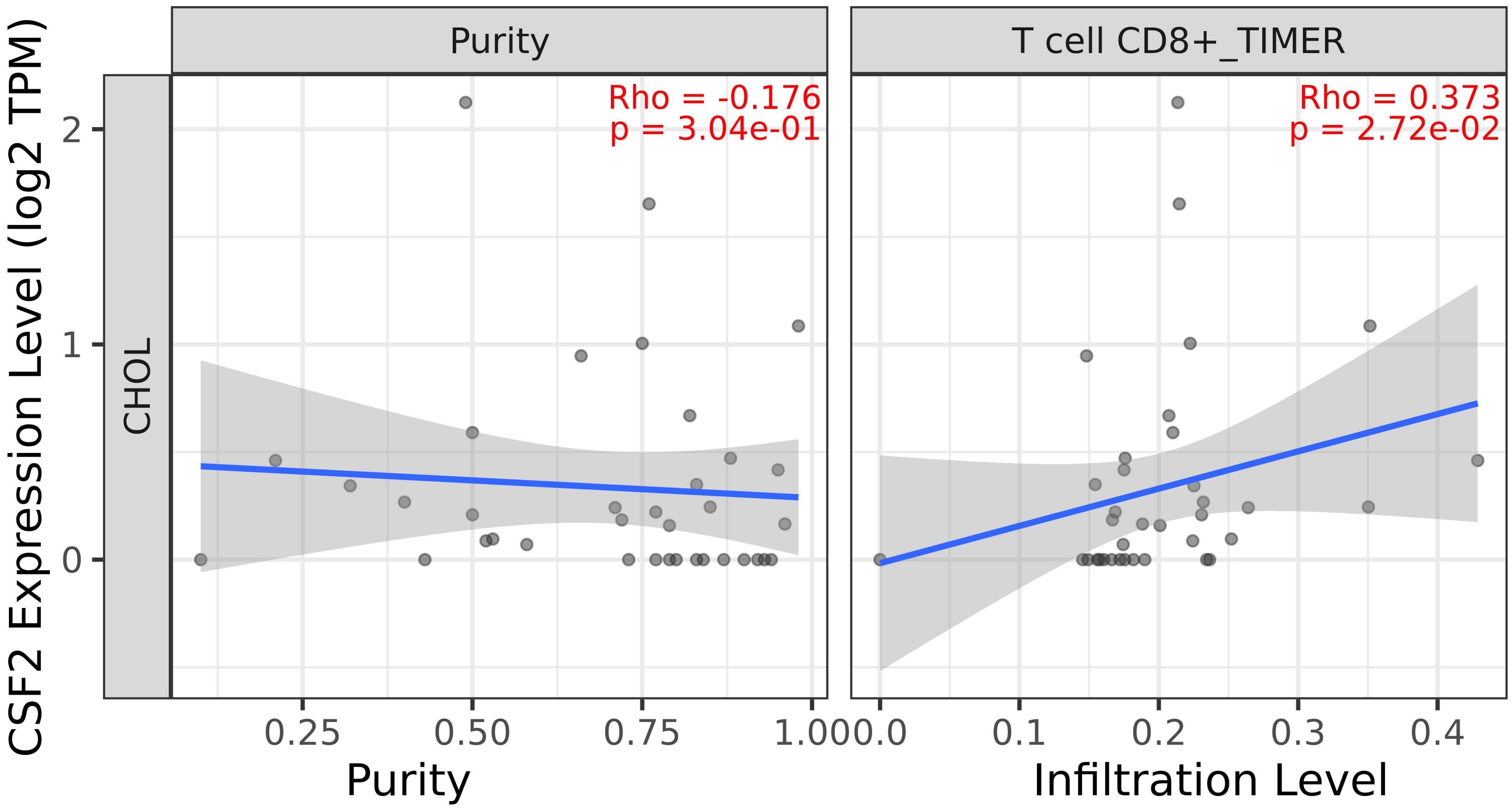

Purity
T cell CD8+_TIMER
CHOL
Rho = -0.176
p = 3.04e-01
Rho = 0.373
p = 2.72e-02
CSF2 Expression Level (log2 TPM)
2
1
0
0.25
0.50
0.75
1.00
0.0
0.1
0.2
0.3
0.4
Purity
Infiltration Level

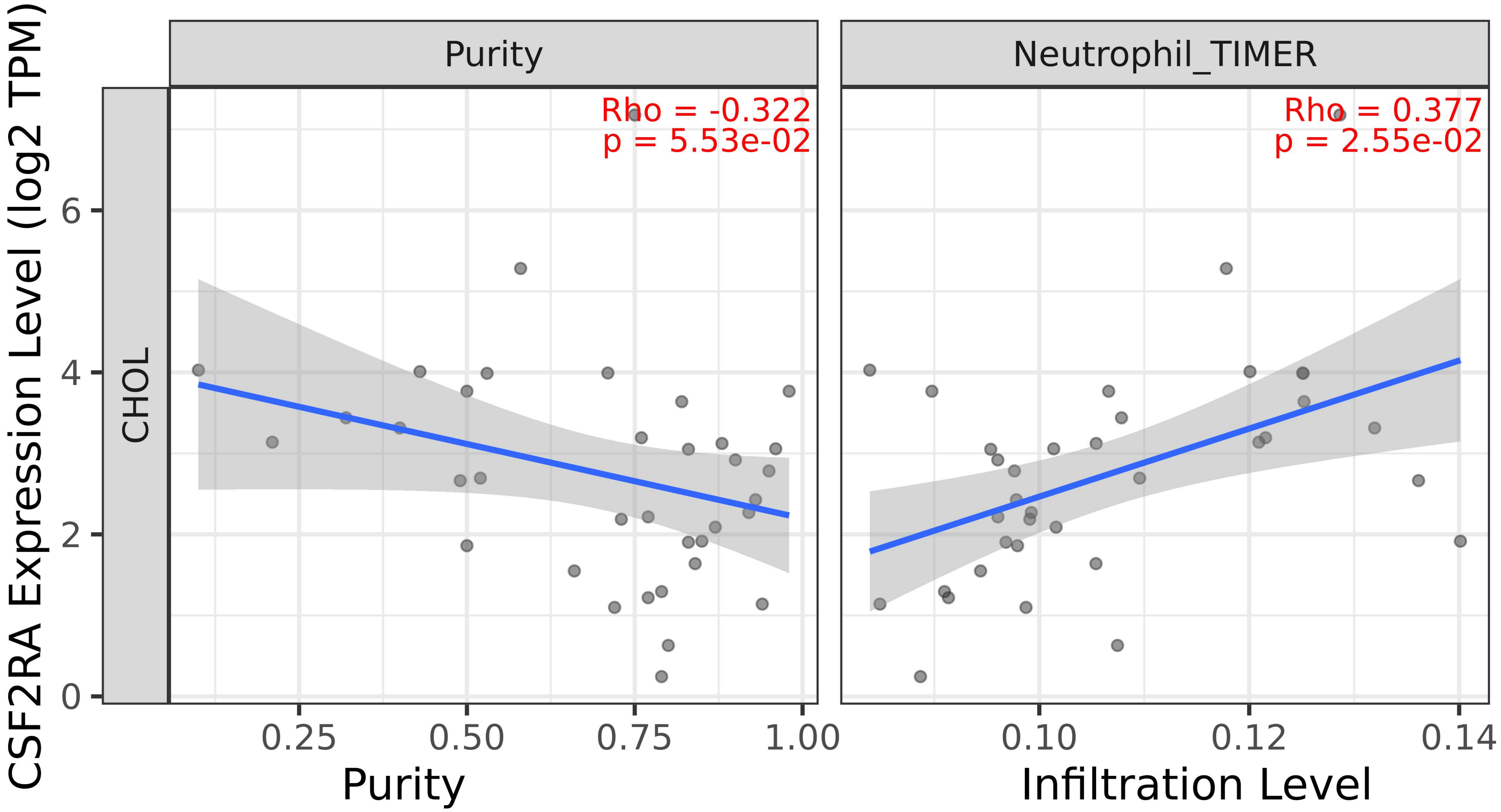
Purity
Neutrophil_TIMER
CHOL
Rho = -0.322
p = 5.53e-02
Rho = 0.377
p = 2.55e-02
CSF2RA Expression Level (log2 TPM)
0
2
4
6
0.25
0.50
0.75
1.00
0.10
0.12
0.14
Purity
Infiltration Level

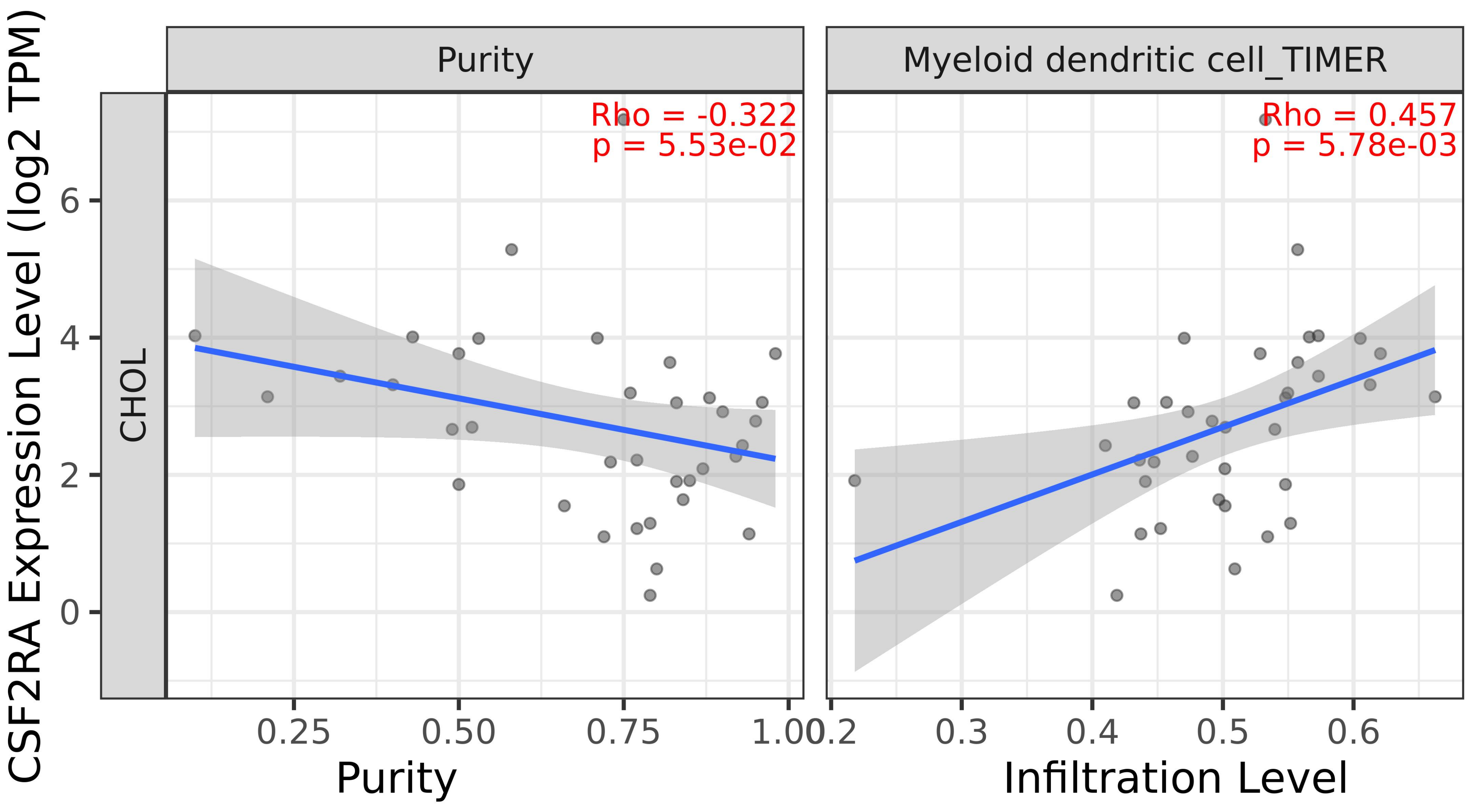

Purity
Myeloid dendritic cell_TIMER
Rho = -0.322
p = 5.53e-02
Rho = 0.457
p = 5.78e-03
CHOL
CSF2RA Expression Level (log2 TPM)
6
4
2
0
0.25
0.50
0.75
1.00
0.2
0.3
0.4
0.5
0.6
Purity
Infiltration Level

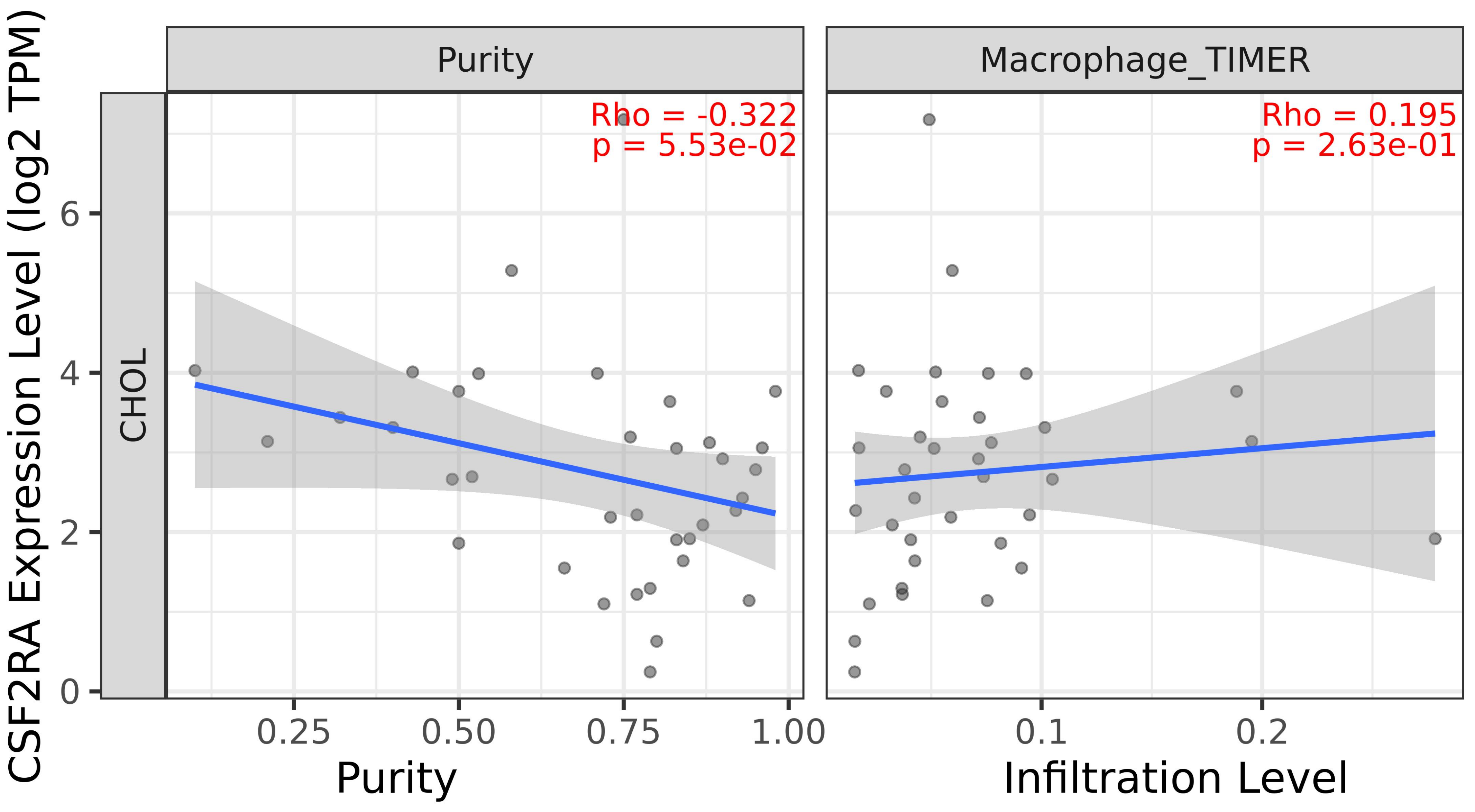

Purity
Macrophage_TIMER
Rho = -0.322
p = 5.53e-02
Rho = 0.195
p = 2.63e-01
CHOL
CSF2RA Expression Level (log2 TPM)
0
2
4
6
0.25
0.50
0.75
1.00
0.1
0.2
Purity
Infiltration Level

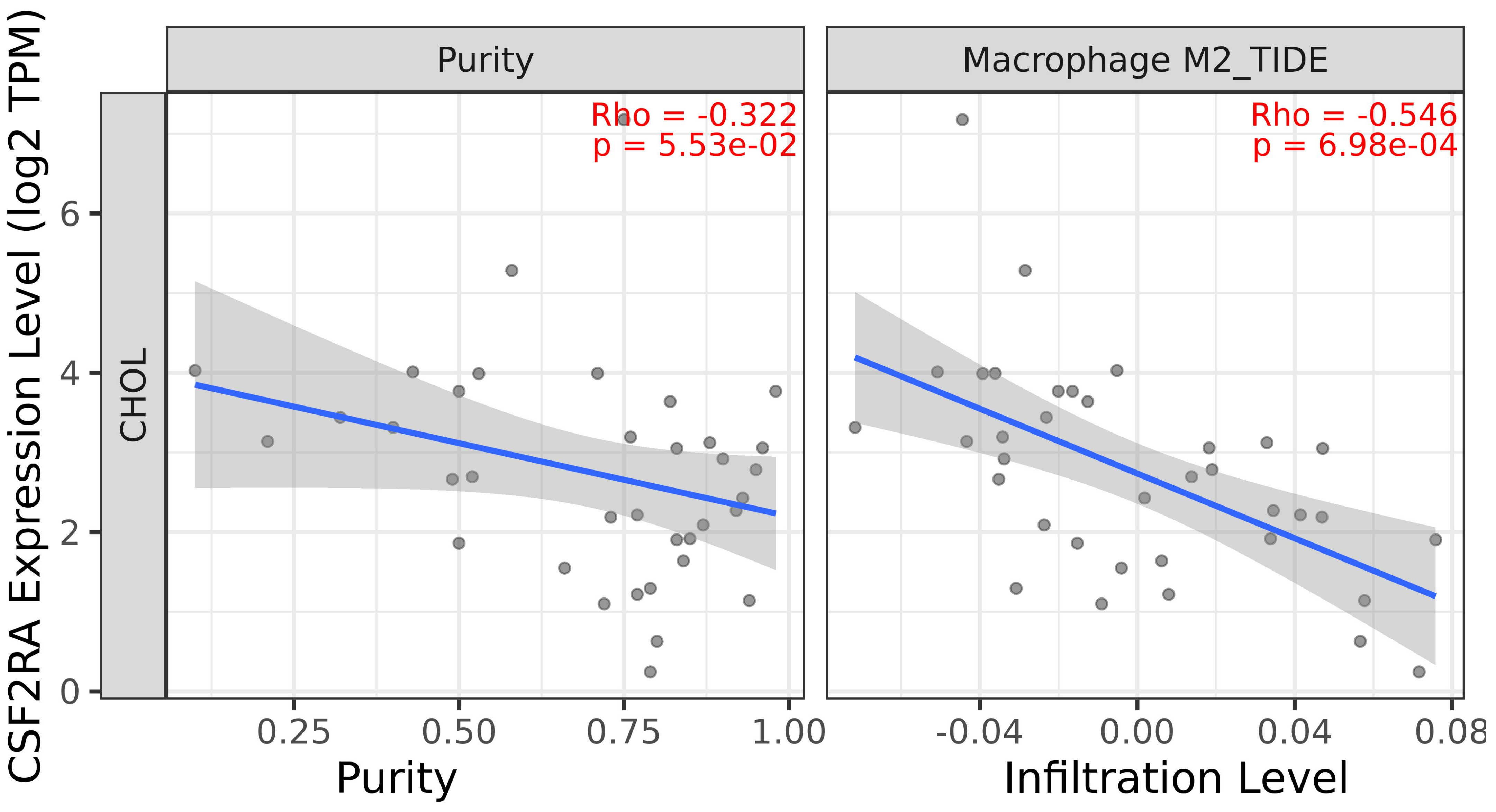
Purity
Macrophage M2_TIDE
CHOL
Rho = -0.322
p = 5.53e-02
Rho = -0.546
p = 6.98e-04
CSF2RA Expression Level (log2 TPM)
0
2
4
6
0.25
0.50
0.75
1.00
-0.04
0.00
0.04
0.08
Purity
Infiltration Level

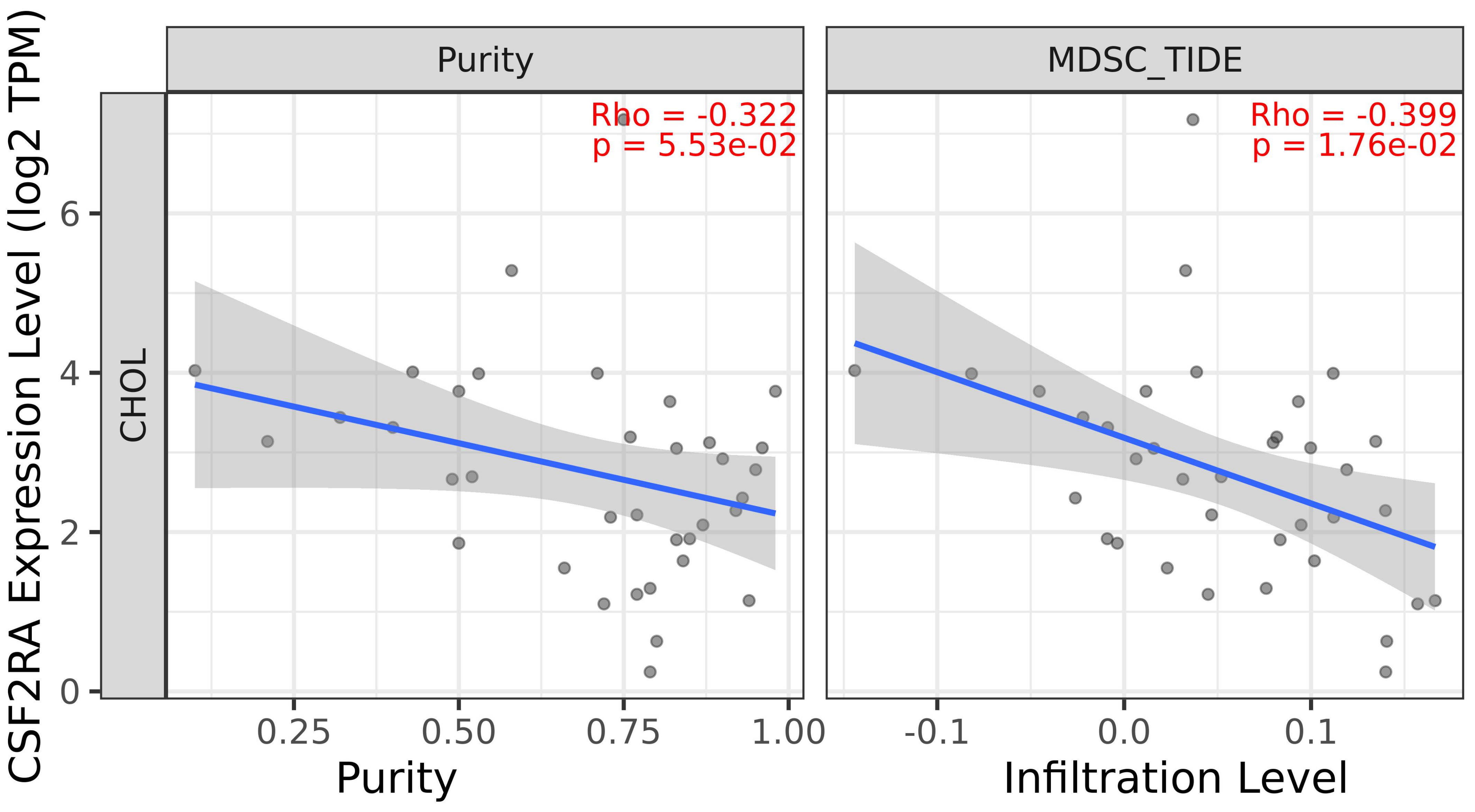

Purity
MDSC_TIDE
Rho = -0.322
p = 5.53e-02
Rho = -0.399
p = 1.76e-02
CHOL
CSF2RA Expression Level (log2 TPM)
0
2
4
6
0.25
0.50
0.75
1.00
-0.1
0.0
0.1
Purity
Infiltration Level

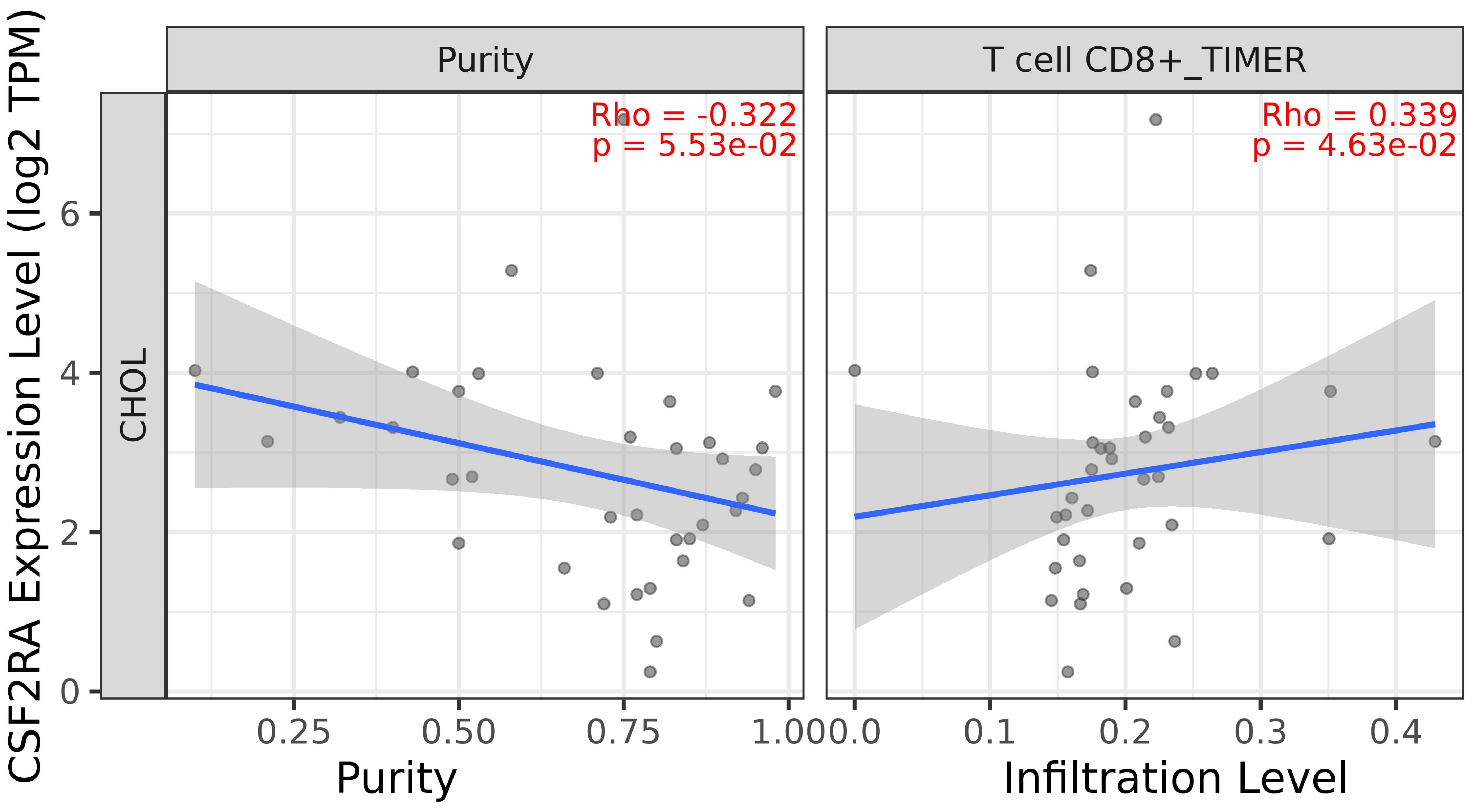

Purity
T cell CD8+_TIMER
CHOL
Rho = -0.322
p = 5.53e-02
Rho = 0.339
p = 4.63e-02
CSF2RA Expression Level (log2 TPM)
0
2
4
6
0.25
0.50
0.75
1.00
0.0
0.1
0.2
0.3
0.4
Purity
Infiltration Level
